# Large Lung Volumes Delay the Onset of the Physiological Breaking Point During Simulated Diving

**DOI:** 10.3389/fphys.2021.731633

**Published:** 2021-09-29

**Authors:** Paul F. McCulloch, B. W. Gebhart, J. A. Schroer

**Affiliations:** ^1^Department of Physiology, College of Graduate Studies, Midwestern University, Downers Grove, IL, United States; ^2^Chicago College of Osteopathic Medicine, Midwestern University, Downers Grove, IL, United States; ^3^Physical Therapy Program, College of Health Sciences, Midwestern University, Downers Grove, IL, United States

**Keywords:** face immersion, human diving response, lung volume, physiological breaking point, chemoreceptor stimulation

## Abstract

During breath holding after face immersion there develops an urge to breathe. The point that would initiate the termination of the breath hold, the “physiological breaking point,” is thought to be primarily due to changes in blood gases. However, we theorized that other factors, such as lung volume, also contributes significantly to terminating breath holds during face immersion. Accordingly, nine naïve subjects (controls) and seven underwater hockey players (divers) voluntarily initiated face immersions in room temperature water at Total Lung Capacity (TLC) and Functional Residual Capacity (FRC) after pre-breathing air, 100% O_2_, 15% O_2_ / 85% N_2_, or 5% CO_2_ / 95% O_2_. Heart rate (HR), arterial blood pressure (BP), end-tidal CO_2_ (etCO_2_), and breath hold durations (BHD) were monitored during all face immersions. The decrease in HR and increase in BP were not significantly different at the two lung volumes, although the increase in BP was usually greater at FRC. BHD was significantly longer at TLC (54 ± 2 s) than at FRC (30 ± 2 s). Also, with each pre-breathed gas BHD was always longer at TLC. We found no consistent etCO_2_ at which the breath holding terminated. BDHs were significantly longer in divers than in controls. We suggest that during breath holding with face immersion high lung volume acts directly within the brainstem to actively delay the attainment of the physiological breaking point, rather than acting indirectly as a sink to produce a slower build-up of PCO_2_.

## Introduction

The diving response, which is present in all mammals, including humans, includes a parasympathetically-mediated bradycardia that decreases cardiac output, a sympathetically-mediated increase in arterial blood pressure through selective restriction of blood flow to non-vital tissues, and apnea (for reviews see Gooden, 1994; Foster and Sheel, 2005; Lindholm and Lundgren, 2009; Castellini, 2012; Fitz-Clarke, 2018). Although voluntary breath holding in air can produce cardiovascular responses similar to that of the diving response, stimulation of trigeminal receptors on the face is necessary for full development of the diving response (Manley, 1990; Gooden, 1994; Andersson et al., 2000). The initiation of the diving response with immersion of the face into water, especially cold water, is well documented and is consistent with research in other animals and in humans with full body immersion (Kawakami et al., 1967; Moore et al., 1972; Fitz-Clarke, 2018).

The duration of a breath hold in either naïve subjects or experienced divers can be quite variable. Breath hold durations (BHDs) typically vary from 20 to 270 s (Lin, 1982), although voluntary breath holds during submergence reaching 11 min have been reported (Fitz-Clarke, 2018). After a voluntary inhibition of breathing and immersion of the face into water, a person can easily hold their breath, called the “easy-going” phase (see Lin, 1982). Throughout this time a physiological urge to breathe develops, primarily through changes in blood gases (i.e., increases in PaCO_2_ and/or decreases in PaO_2_). The point where these physiological parameters have built up to levels that initiate the termination of the breath hold is called the “physiological breaking point.” At the physiological breaking point there is an onset of involuntary ventilatory activity, such as an increase in diaphragmatic activity. Some subjects can continue to hold their breath past the physiological breaking point by consciously suppressing their urge to breathe, and overcoming the discomfort of hypoxia, hypercapnia, and acidosis, etc. (Fitz-Clarke, 2018). This phase is called the “struggle phase,” and may involve psychological and/or motivational factors that inhibit the termination of the breath hold. Strong involuntary breathing movements during the “struggle phase” may also increase brain blood flow (Dujic et al., 2009) and splenic contraction (Palada et al., 2008). The end of the struggle phase is the “conventional breaking point,” where the breath holding can no longer be continued, and the subject voluntarily terminates their breath hold, reemerges from the face submersion, and resumes normal ventilatory activity.

The physiological breaking point is sharply defined by chemical status (Lin, 1987, 1988). However, in addition to changes in blood gases, lung volume may also play a role in determining the duration of the easy-going phase. Both the easy going phase and total BHDs are longer when breath holding at total lung capacity (TLC) than at functional residual capacity (FRC) (Andersson and Schagatay, 1998). This may be because, with a larger lung volume, it could take longer for PaCO_2_ and PaO_2_ to reach levels that signal the physiological breaking point (Andersson and Schagatay, 1998), an effect perhaps aided through a potentiation of diving bradycardia (Manley, 1990). Alternatively, the level of lung inflation may provide afferent signals, through pulmonary stretch receptor activity, that could directly constitute a physiological signal that affects the onset of the physiological breaking point. Accordingly, we designed the present study to determine whether lung volume *per se* can affect the duration of the easy-going phase in either untrained or experienced divers (members of an underwater hockey team). Also, since chemical status can affect BHD, we had subjects pre-breath different gas mixtures to enhance or diminish chemoreceptor activity during the breath holds. We hypothesized that activation of pulmonary stretch receptors at large lung volumes act to prolong the attainment of the physiological breaking point.

## Materials and Methods

### Subjects

This study was approved by the Midwestern University Institutional Review Board (IRB) prior to subject enrollment. All subjects gave informed consent and understood the procedures, risks, and voluntary nature of the study.

Subjects (ages 21–42) were recruited from Midwestern University in Downers Grove, IL (“controls”: *N* = 9; 4M, 5F) and a local underwater hockey club (“divers” *N* = 7; 4M, 3F). All subjects were healthy, non-smokers without apparent cardiovascular or respiratory disease and not taking any medication that would interfere with study parameters. In addition, subjects abstained from caffeine use on the day of testing. No adverse experiences were reported.

### Study Procedures

A total of eight trials were performed at two lung volumes—four at TLC and four at FRC. Before each breath hold trial, subjects were asked to breathe one of the following four gas mixtures to alter chemoreceptor activity. Standard Air (21% O_2_) was the control gas; 100% O_2_ was chosen to decrease peripheral chemoreceptor activity; 15% O_2_ / 85% N_2_ was chosen to increase peripheral chemoreceptor activity; and 5% CO_2_ / 95% O_2_ was chosen to increase central chemoreceptor activity and decrease peripheral chemoreceptor activity. The assignment of each gas was random, predetermined, and conducted in a single-blind fashion—subjects were blinded to the gas mixture administered before each trial.

Two practice trials were performed prior to recorded testing to acclimate the subjects to the testing format. Four trials with each of the four gas mixtures were conducted at TLC followed by four additional trials at FRC.

The subjects were seated and breathed the appropriate gas mixture for 5 min according to a randomization list, *via* a disposable facemask. After 3 min. of breathing the gas, an expired CO_2_ measurement was obtained by having the subject take a full breath, remove the face mask without releasing any air and blow into an end-tidal CO_2_ (etCO_2_) analyzer (see below). The subject then replaced the facemask without taking a breath and continued to breathe the gas mixture for an additional 1 min. At the end of the last minute the subject was given a verbal cue to halt their breathing at either TLC or FRC, remove the facemask, and immediately immerse their face in a basin of room temperature water. To ensure full stimulation of the diving response the forehead and eye areas were required to be submerged.

Subjects were instructed to hold their breath as long as comfortably possible, but to end their breath hold after they felt the urge to breathe. They were also instructed to refrain from hyperventilation before the breath hold, to refrain from movements of the chest or other skeletal muscles, including swallowing movements, during the breath hold, and not to release any air into the water. At the end of the breath hold, the subject removed their face from the water and immediately provided a second etCO_2_ measurement before taking a breath of room air. They were allowed to breathe normally once this measurement was obtained. Five-minute rest periods separated each trial.

Heart Rate [HR, in beats per minute (BPM)] and arterial blood oxygen saturation (SaO_2_, in percent saturation) were recorded throughout each trial using a cardiosync pulse oximeter (Noni^TM^n Medical, Model 8700) with toe clip sensor and contact thermal printer printing continuously in real time. The oximeter had a stated accuracy of ± 2% of full scale in the range of 70–100% SaO_2_. Systolic and diastolic blood pressure were measured using an automatic digital blood pressure monitor with printer (Omron^®^, Model HEM-703CP), and were used to calculate mean arterial blood pressure (BP, in mm Hg). EtCO_2_ (in percent) was measured using a CO_2_ analyzer (BIOPAC CO_2_100C). BHD (in s) was recorded using the touch-print feature of the oximeter. Lastly, an elastic chest band recorded chest excursion to evaluate lung volume and extraneous chest movements during each trial (BIOPAC TSD101B).

Blood pressure was obtained once approximately 30 s prior to the breath hold and once during the breath hold. However, BP readings may not have been obtained during the trials that lasted less than 30 s. EtCO_2_ was measured 1 min prior to the breath hold and again immediately after the breath hold was terminated. SaO_2_ and HR were measured throughout the 3 min prior to the breath hold until completion of the second CO_2_ measurement. Data points were collected at 10-s intervals as well as at the timepoints of breath hold initiation and termination. To minimize recording errors, the subjects were instructed to keep their foot still while the toe clip was in place. Water and air temperatures were recorded prior to the first trial. Finally, all subjects were questioned about adverse experiences.

### Statistical Analysis

All data is presented as mean ± standard error (SE). Statistical comparisons were made with a computer statistical package (SigmaStat v14) and *p* < 0.05 was used as the level of significance. For both the controls and divers, data for males and females were not significantly different, and so were grouped together. For all data, three-way ANOVAs compared controls vs. divers, TLC vs. FRC, and the four gases pre-breathed before the face immersions. Two-way ANOVAs compared TLC vs. FRC and the four gases pre-breathed before the face immersions for HR and SaO_2_ at specific time points during the face immersions. One-way repeated measures ANOVAs were used to compare HR and SaO_2_ during the face immersion to the control value before the face immersion. Even if the BHD lasted longer than 35 s, statistical analysis on HR and SaO_2_ was only done on the first 35 s of the face immersion to facilitate statistical comparisons between groups. If statistical significance was reached during comparisons, it was then followed by *post hoc* testing (Tukey’s multiple comparison procedure or Dunnett’s multiple comparison vs. control) to determine which values were different from the others. *T*-tests were used to compare BP and etCO_2_ before and after the face immersions.

## Results

Initial analysis combines data from control and divers to determine the effects of lung volume and pre-existing chemoreceptor stimulation on HR, BP, SaO_2_, etCO_2_ and BHD responses to face immersion. We then subsequently analyze data between the control and divers to determine whether breath hold diving experience affects any of these parameters.

**FIGURE 1:**
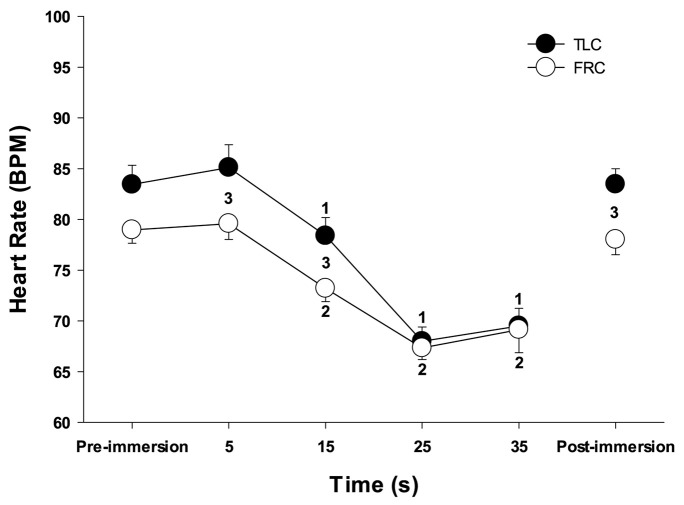
Face immersion into room temperature water produced a significant decrease in HR when breath holding at both TLC and FRC. 1, HR is significantly less than TLC pre-immersion HR; 2, HR is significantly less than FRC pre-immersion HR; 3, TLC HR is significantly greater than FRC HR.

Face immersion into room temperature water produced a significant decrease in HR when breath holding at both TLC (*F* = 80.109, *p* < 0.001) and FRC (*F* = 69.145, *p* < 0.001) (Figure 1). HRs were significantly higher when breath holding at TLC vs. FRC at pre-immersion (*F* = 5.230, *p* = 0.024), 5 s (*F* = 9.734, *p* = 0.002), and 15 s (*F* = 17.522, *p* < 0.001) (Figure 1). However, the magnitude of the bradycardias was not significantly different when breath holding at either TLC or FRC. Chemoreceptor stimulation by breathing different gases before face immersion had little effect on the decrease in HR (Table 1). There were no significant differences in the HR responses between the four TLC face immersions, nor between the four FRC face immersions. Additionally, for each of the four gases, there were no significant differences in the HR responses between TLC and FRC face immersions.

**TABLE 1:** Chemoreceptor stimulation by breathing different gases before face immersion did not affect the HR response (presented as HR ± SE) to face immersion.

**Time**	**AIR**		**15% O_2_**		**100% O_2_**		**5% CO_2_/95% O_2_**	
	**TLC**	**FRC**	**TLC**	**FRC**	**TLC**	**FRC**	**TLC**	**FRC**
Pre-immersion	86 ± 4	81 ± 3	85 ± 4	78 ± 2	81 ± 3	79 ± 3	82 ± 4	78 ± 3
5 s	87 ± 4	82 ± 4	88 ± 6	79 ± 3	81 ± 4	80 ± 3	84 ± 4	78 ± 3
15 s	77 ± 2^1^	75 ± 3	76 ± 3	71 ± 3^2^	78 ± 3	73 ± 3	82 ± 6	74 ± 2
25 s	69 ± 3^1^	69 ± 3^2^	67 ± 2^1^	66 ± 22	68 ± 4^1^	66 ± 2^2^	67 ± 3^1^	67 ± 2^2^
35 s	70 ± 4^1^	70 ± 6^2^	69 ± 3^1^	69 ± 6^2^	69 ± 3^1^	70 ± 3^2^	70 ± 4^1^	69 ± 4^2^
Post-immersion	82 ± 3	76 ± 3	83 ± 3	76 ± 4	85 ± 3	81 ± 2	84 ± 3	78 ± 3

*There were no significant differences between the four TLC face immersions, or between the four FRC face immersions.*

*Additionally, for each of the four gases, there were no significant differences between the TLC and FRC face immersions.*

*1, HR significantly less than TLC pre-immersion HR; 2, HR significantly different than FRC pre-immersion HR.*

**FIGURE 2:**
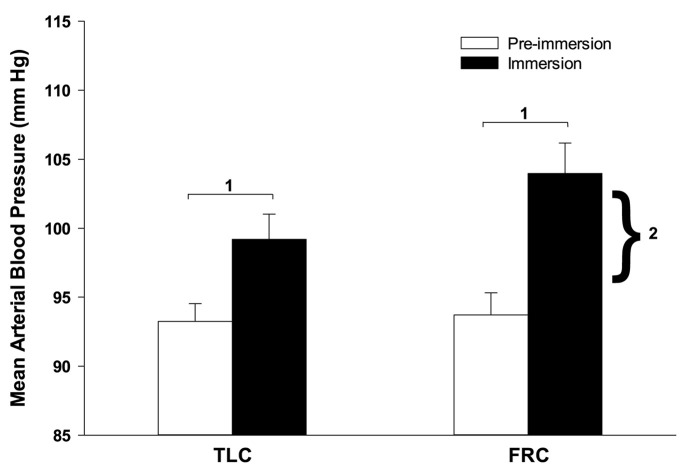
Face immersion into room temperature water produced a significant increase in BP when breath holding at both TLC and FRC. The increase in BP was significantly greater during FRC face immersions than TLC face immersions. 1, immersion BP is significantly greater than pre-immersion BP; 2, the increase in BP at FRC is significantly greater than the increase in BP at TLC.

Face immersion produced a significant increase in BP when breath holding at both TLC (*p* < 0.001) and FRC (*p* < 0.001; Figure 2). BP increased by 6 ± 1 mm Hg when breath holding at TLC, and by a significantly greater 10 ± 2 mm Hg when breath holding at FRC (*p* = 0.028). Chemoreceptor stimulation by breathing different gases before face immersion had little effect on increase in BP (Table 2). There were no significant differences between the BPs for the four TLC face immersions, nor between the BPs for the four FRC face immersions.

**TABLE 2:** Chemoreceptor stimulation by breathing different gases before face immersion did not affect BP response (presented as BP ± SE) to face immersion.

**Time**	**AIR**		**15% O_2_**		**100% O_2_**		**5% CO_2_/95% O_2_**	
	**TLC**	**FRC**	**TLC**	**FRC**	**TLC**	**FRC**	**TLC**	**FRC**
Pre-immersion	89 ± 2	97 ± 5	93 ± 2	93 ± 2	96 ± 4	92 ± 2	94 ± 2	93 ± 2
Immersion	100 ± 3^1^	105 ± 5	98 ± 3	103 ± 4^1^	99 ± 4	102 ± 3^1^	100 ± 4	108 ± 7^1^

*The increase in BP during face immersion was usually greater at FRC than at TLC.*

*There were no significant differences between the BPs for the four TLC face immersions, or between the BPs for the four FRC face immersions.*

*1, Immersion BP significantly greater than pre-immersion BP.*

During face immersion in room temperature water, SaO_2_ had a slight downward trend, but remained greater than 98% in both TLC and FRC face immersions. There were no differences in SaO_2_ between TLC and FRC during face immersion. When pre-breathing 100% O_2_ or 5% CO_2_ / 95% O_2_, SaO_2_ was statistically greater than when pre-breathing air or 15% O_2_ (*F* = 14.628, *p* < 0.001). However, pre-breathing different gases had little effect on SaO_2_ during face immersion, and even when pre-breathing 15% O_2_ before face immersion, SaO_2_ did not fall below 98% during face immersion.

**FIGURE 3:**
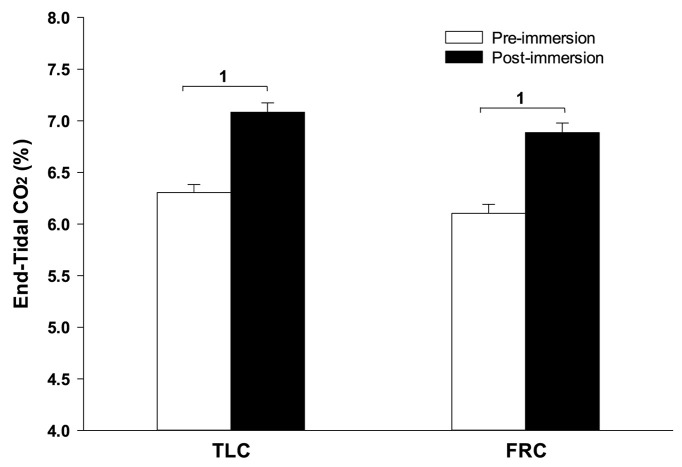
Face immersion into room temperature water produced a significant increase in etCO_2_ when breath holding at both TLC and FRC. 1, Post-immersion etCO_2_ is significantly greater than pre-immersion etCO_2_.

**FIGURE 4:**
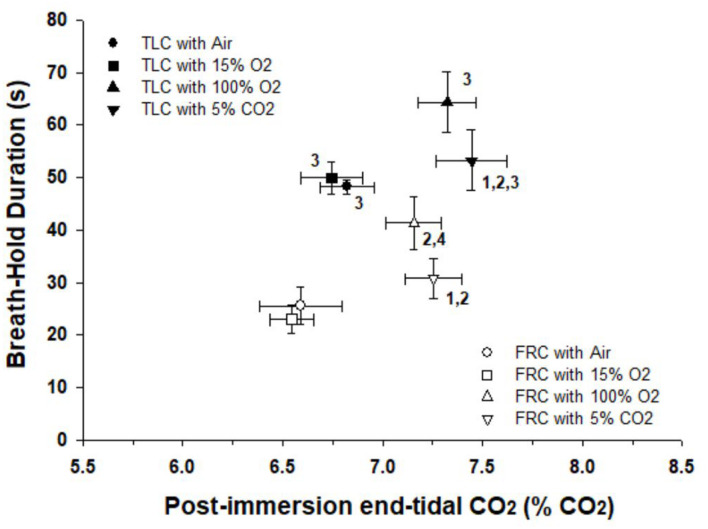
Breath holds after face immersion into room temperature water did not end at a consistent etCO_2_, and BHD was always longer at TLC than at FRC. There were significant differences between the post-immersion etCO_2_’s after pre-breathing different gases before breath holds. Thus there was no consistent etCO_2_ that initiated the termination of the breath holds. For each gas breathed before the breath hold, BHD at TLC was always longer than BHD at FRC. 1, Post-immersion etCO_2_ is significantly greater than post-immersion etCO_2_ after pre-breathing air at that lung volume. 2, Post-immersion etCO_2_ is significantly greater than post-immersion etCO_2_ after pre-breathing 15% O_2_ at that lung volume. 3, BDH at TLC is significantly longer than BHD at FRC after pre-breathing that specific gas. 4, BHD after pre-breathing 100% O_2_ at FRC is significantly longer than BHD after pre-breathing 15% O_2_ at FRC.

During face immersion in room temperature water, etCO_2_ increased significantly from 6.13% ± 0.08 pre-immersion to 6.98% ± 0.09 post-immersion with TLC (*p* < 0.001), and from 5.92% ± 0.08 pre-immersion to 6.81% ± 0.09 post-immersion with FRC (*p* < 0.001) (Figure 3). Pre-breathing different gases did not change the pre-immersion etCO_2_, or the fact that there was a significant increase in etCO_2_ during the immersion (*F* = 10.52, *p* < 0.001). However, there was no consistent end-immersion etCO_2_ at which the breath holds terminated (Figure 4). After pre-breathing 5% CO_2_ / 95% O_2_ post-immersion etCO_2_ (7.24% ± 0.12) was significantly greater than for air (6.66% ± 0.12; *F* = 7.42, *p* = 0.003) or 15% O_2_ (6.59% ± 0.10; *F* = 7.42, *p* < 0.001). Also, after pre-breathing 100% O_2_, the post-immersion etCO_2_ (7.09% ± 0.13) was significantly greater than for air (*F* = 7.42, *p* = 0.028) or 15% O_2_, (*F* = 7.42, *p* = 0.013). In addition, there was no consistent increase in etCO_2_ after which the breath holds terminated. After breath holding at TLC, etCO_2_ increased by 1.21% ± 0.11 after pre-breathing 100% O_2_, which was a significantly greater increase in etCO_2_ (*F* = 7.39, *p* < 0.001) than that seen after pre-breathing air (0.66% ± 0.001; *p* < 0.001) or 15% O_2_ (0.64% ± 0.08; *p* < 0.001).

BHD after face immersion was significantly longer (*F* = 55.18, *p* < 0.001) at TLC (59 ± 3 s) than at FRC (32 ± 2 s), although it is notable that the post-immersion etCO_2_ at TLC and FRC was not significantly different from each other (*F* = 2.248, *p* = 0.137). Additionally, for each gas breathed before the breath hold, BHD when breath holding at TLC was always significantly longer than BHD when breath holding at FRC (air, *p* < 0.001; 15% O_2_, *p* < 0.001; 100% O2, *p* = 0.003; 5% CO_2_ / 95% O_2_, *p* = 0.003) (Figure 4).

**FIGURE 5:**
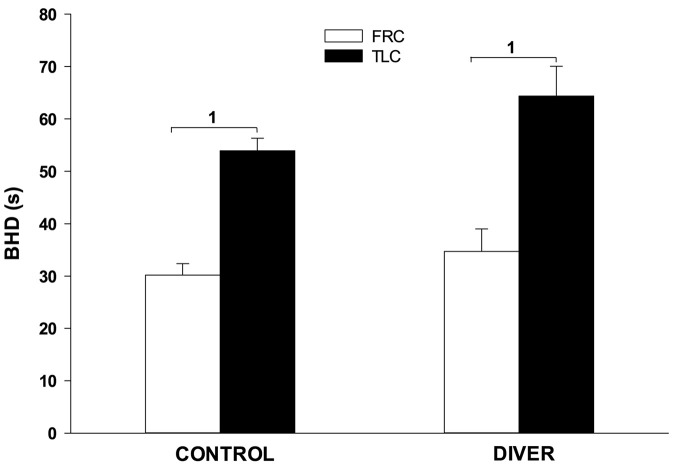
Face immersion into room temperature water at TLC significantly increases BHD in both controls and divers. 1, BHD is significantly longer at TLC vs. FRC.

Divers had a qualitatively similar cardiovascular diving response to the control subjects, and the differences between TLC and FRC face immersions that were seen in controls were also seen in divers. The pre-immersion HR of divers was significantly lower than the pre-immersion HR of controls (66 ± 1 for divers vs. 81 ± 1 for controls; *F* = 67.702, *p* < 0.001). Consequently, the face immersions HRs were significantly lower in divers than in controls (5 s, *F* = 54.868, *p* < 0.001; 15 s, *F* = 66.178, *p* < 0.001; 25 s, *F* = 100.817, *p* < 0.001; 35 s, *F* = 88.106, *p* < 0.001). However, the magnitude and time course of the bradycardias were similar between divers and controls. During face immersion divers showed a significant increase in BP (*p* < 0.001) that was similar in magnitude to that of controls (8 ± 2 mm Hg for controls vs. 7 ± 1 mm Hg for divers). As in controls, SaO_2_ had a slight downward trend during the breath holds in divers but did not decrease below 98% for any of the gases that were pre-breathed. The pre-immersion etCO_2_ of divers was significantly lower than that of controls (5.81% ± 0.10 for divers vs. 6.02% ± 0.06 for controls; *F* = 12.65, *p* < 0.001), although the end-immersion etCO_2_’s were not significantly different. Consequently, the increase in etCO_2_ during the face immersions was significantly greater for divers than for controls (0.97% ± 0.05 for divers vs. 0.79% ± 0.06 for controls; *F* = 6.38, *p* < 0.013). BHD in divers (50 ± 4 s) was significantly longer than BHD in controls (42 ± 2 s) (*F* = 4.34; *p* = 0.039), but not when specifically comparing at either TLC or FRC. In addition, for divers BHD at TLC (64 ± 6 s) was significantly longer (*p* < 0.001) than at FRC (35 ± 4 s). For controls BHD at TLC (54 ± 2 s) was significantly longer (*p* < 0.001) than at FRC (30 ± 2 s) (Figure 5).

## Discussion

The most important finding from this study is that lung volume, and therefore presumably pulmonary stretch receptor activity, is an important physiological factor that contributes to the onset of the physiological breaking point during voluntary breath holding in room temperature water. BHD at TLC was always significantly longer than BHDs at FRC, even though the cardiovascular responses to face immersion at the two lung volumes were similar. Additionally, BHDs at TLC were always longer regardless of which gas was breathed before the breath hold or whether the subject had previous breath holding experience. Although PCO_2_ is the most important factor that contributes to the onset of the physiological breaking point (Lin et al., 1974; Fitz-Clarke, 2018), we found that there was no consistent etCO_2_ at which breath holds terminated.

### Human Diving Response

Like previous studies investigating the human diving response, we found that face immersion in room temperature water produced bradycardia and an increase in blood pressure (Gooden, 1994; Foster and Sheel, 2005; Lindholm and Lundgren, 2009; Fitz-Clarke, 2018). Changing chemoreceptor stimulation by pre-breathing different gases before face immersion had little effect on the development of the bradycardia or the increase in BP. The HR response to breath holding after face immersion was similar at either TLC or FRC. Similar results were found by Andersson and Schagatay (1998) who reported that lung volume [60, 85, or 100% of Vital Capacity (VC)] does not appreciably change HR, skin capillary blood flow or blood pressure responses to breath holding. However, unlike Andersson and Schagatay (1998), we found that there was a greater increase in BP when breath holding at lower lung volumes (FRC) than at higher lung volumes (TLC). The intensification of peripheral vasoconstriction when breath holding at FRC would presumably lead to greater oxygen conservation during the breath hold (Andersson et al., 2000). Instead, however, BHD at FRC was significantly shorter than BHD at TLC.

### Chemoreceptor Stimulation and the Physiological Breaking Point

The physiological breaking point signals the end of the easy-going phase of a voluntary breath hold. When physiological variables build up to the point where a signal to terminate the breath hold has been reached, the breath hold can only be continued through voluntary suppression of the drive to terminate the breath hold (Lin et al., 1974; Hentsch and Ulmer, 1984). The primary physiological variable that is thought to determine the physiological breaking point is an increase in chemoreceptor activity through an increase in PCO_2_ or a decrease in PO_2_ (Lin et al., 1974; Parkes, 2006). When breath holding with oxygen without face submersion, the physiological breaking point is reached when alveolar PCO_2_ reaches 48–54 mm Hg (Lin et al., 1974; Parkes, 2006), although the PCO_2_ will be lower if there is simultaneous hypoxia (Lin, 1987).

EtCO_2_ increased significantly during face immersions, and we found that breath holds at both TLC and FRC ended at an etCO_2_ of about 7%. Andersson and Schagatay (1998) found that breath holds at 60%, 85% and 100% of VC ended at an etCO_2_ of approximately 7.4%. When subjects pre-breathed different gases before breath holding, we found no post-immersion etCO_2_ at which all face immersions ended. Rather, pre-breathing 100% O_2_ or 5% CO_2_ / 95% O_2_ at FRC, or 5% CO_2_ / 95% O_2_ at TLC, significantly increased the post-immersion etCO_2_. Also, the difference between the pre-immersion etCO_2_ and post-immersion etCO_2_ was increased after pre-breathing 100% O_2_, at least at TLC. These data suggest that there is a synergistic effect between CO_2_ central chemoreceptor stimulation and O_2_ peripheral chemoreceptor stimulation (Fowler, 1954; Lin et al., 1974). Breathing a high O_2_ gas permits breath holds to terminate at higher etCO_2_’s. However, there is no absolute etCO_2_ that, when reached, terminates a breath hold.

In the present study, BHDs were so short that SaO_2_ decreased very little during face immersion. Hemoglobin remained above 98% saturated with oxygen, even after pre-breathing hypoxic gas. Consequently it was unlikely that arterial chemoreceptors were greatly stimulated through increasing hypoxemia during any of these breath holds. Therefore the peripheral chemoreflex, by itself, probably did not contribute to the cardiovascular (i.e., HR and BP) changes observed during the breath holds, or to the attainment of the physiological breaking point. However, even though oxygen saturations did not decrease substantially during the breath holds, PO_2_ had a synergistic effect with PCO_2_, as evidenced by significant differences in post-immersion etCO_2_ after pre-breathing different gases (Figure 4).

### Lung Volume and the Physiological Breaking Point

Although beginning a breath hold at either TLC or FRC did not appreciably change the cardiovascular responses to breath holding, breath holding at either TLC or FRC did significantly change BHD. BHDs were always longer at TLC than at FRC, even when subjects pre-breathed different gases. Andersson and Schagatay (1998) found that larger lung volumes increased the duration of both the easy-going phase and total breath holding duration. It is thought that a larger lung volume provides a sink that allows a build-up of CO_2_ and/or depletion of O_2_, thus allowing for a longer BHD (Sterba and Lundgren, 1985, 1988). However, even though there were significant differences in etCO_2_’s after pre-breathing different gases, there were no significant differences in BHD for the four face immersions at each of the two lung volumes (Figure 4). This suggests that lung volume, in addition to etCO_2_, is an important factor in determining physiological breaking point and breath holding capability.

The effect of lung volume on attainment of the physiological breaking point could stem from slowly adapting pulmonary stretch receptor (SAR) afferent activity. With greater expansion of the lungs with greater lung volumes, SAR activity will increase (Sant’Ambrogio, 1982; Schelegle and Green, 2001). When lung inflation is maintained, SARs characteristically show a long-lasting and sustained discharge (Sant’Ambrogio, 1982; Schelegle and Green, 2001). When breath holding at a large lung volume without face immersion, a Breuer–Hering like reflex may act to prolong maximal breath hold duration (Muxworthy, 1951; Chapin, 1955). We suggest that SAR activity during voluntary breath holding with face immersion at large lung volumes acts within the brainstem central rhythm generator to actively prolong phase II expiration and/or to inhibit generation of inspiration (D’Angelo and Agostoni, 1975; St. John and Zhou, 1990; Sant’Ambrogio and Sant’Ambrogio, 1991). The result of the SAR activity would be to extend the duration of the easy-going phase of a breath hold by suppressing attainment of the physiological breaking point that primarily results from an increase in PCO_2_. Thus at large lung volumes, inhibition of breath hold termination would occur due to neuronal interactions within the brainstem central rhythm generator, rather than due to a slower build-up of PCO_2_ within the lungs. Additionally, strong involuntary breathing movements are less frequent at high lung volumes, possibly explaining why higher lung volumes allows for longer BHDs (Fitz-Clarke, 2018).

Other explanations of the direct effect lung volume plays on BHD could be that the prolonged absence of SAR activity when breath holding at low lung volumes is, in itself, a respiratory stimulus that leads to the termination of the breath hold and reestablishment of eupnea (Fowler, 1954; Godfrey and Campbell, 1968, 1969). At low lung volumes there would be neither rhythmic nor static SAR afferent activity, because with eupnic breathing, and thus at lung volumes near to FRC, there is normally no SAR activity in humans (Sant’Ambrogio and Sant’Ambrogio, 1991). Additionally, the absence of any SAR input due to the voluntarily initiated apnea may act to terminate a breath hold (Mithoefer, 1959; Delapille et al., 2001). A technique that experienced breath hold divers sometimes use to prolong BHD is to engage in fictive breathing movements by pumping the thoracic cavity while keeping the glottis closed [personal observations; (Lin, 1982)]. This would have the effect of changing lung volume, and thus provide SAR input to the brainstem, even if no fresh air reaches the lungs. Rebreathing during a breath hold extends BHD, even though arterial blood gases do not improve (Fowler, 1954; Godfrey and Campbell, 1968, 1969). Another technique used to prolong breath holds is to open the epiglottis and swallow. Although this would not change lung volume or SAR activity, it would provide a rhythmic input into brainstem respiratory centers. However, swallowing or taking a “false breath” while breath holding reduces the magnitude of diving bradycardia (Gandevia et al., 1978), and involuntary breathing movements during breath holding are thought to be too small to influence the diving response (Andersson and Schagatay, 1998).

### Effect of Previous Breath Holding Experience

The experienced divers in our study were members of a local underwater hockey club. Qualitatively, the diving response of divers was similar to that of controls. However, the BHD of divers was significantly longer than that of controls, although not as long as that seen in experienced underwater hockey or rugby players (Davis et al., 1987; Schagatay and Andersson, 1998), or synchronized swimmers (Oldridge et al., 1978; Bjurström and Schoene, 1987). However, our divers were recreational underwater hockey players, rather than national caliber players as in the study by Davis et al. (1987). Additionally, through our instructions to them, divers ended their face immersions at or near their physiological breaking points, rather than near to their conventional breaking points. During the struggle phase of breath hold diving, the physiological drive to terminate the breath hold is consciously suppressed. Breath holding experience or apnea training can prolong total BHD by delaying the conventional breaking point (Davis et al., 1987; Schagatay et al., 1999, 2000). Thus, with our instructions to our subjects to end their breath holds when they felt the urge to breath and not to use fictive breathing techniques, we negated the training effects that could have given the trained divers a breath holding “advantage”.

### Conclusion

We conclude many factors bring about the physiological breaking point during breath holding. These include increased PaCO_2_ (as measured by etCO_2_), although there is no absolute etCO_2_ or change in etCO_2_ that, when reached, will initiate the termination of the breath hold. We also conclude that high lung volume, or more specifically the increased pulmonary stretch receptor activity at high lung volume, is an important factor that contributes to the physiological breaking point. We suggest that high lung volume acts centrally to inhibit the termination of breath holds, and thus prolong the duration of the easy-going phase of voluntary breath holding. We also conclude that increased arterial chemoreceptor activity due to the development of hypoxia during the breath hold plays a limited, if any, role in terminating most short duration breath holds. Finally, we conclude that previous breath holding experiences increases breath hold durations without substantially changing cardiorespiratory (HR, BP SaO_2_, and etCO_2_) responses to face immersion.

## Data Availability Statement

The raw data supporting the conclusions of this article will be made available by the authors, without undue reservation.

## Ethics Statement

The studies involving human participants were reviewed and approved by Midwestern University Institutional Review Board. The patients/participants provided their written informed consent to participate in this study.

## Author Contributions

PM and BG conceptualized and planned the study, formulated research protocols, and obtained IRB approval. BG and JS collected data. PM performed statistical analysis, created figures, and wrote the manuscript. All authors contributed to the article and approved the submitted version (except JS).

## Conflict of Interest

The authors declare that the research was conducted in the absence of any commercial or financial relationships that could be construed as a potential conflict of interest.

## Publisher’s Note

All claims expressed in this article are solely those of the authors and do not necessarily represent those of their affiliated organizations, or those of the publisher, the editors and the reviewers. Any product that may be evaluated in this article, or claim that may be made by its manufacturer, is not guaranteed or endorsed by the publisher.
